# Module intervention to improve involvement and practices of fathers towards infant and young child feeding (IYCF) in Coastal South India - a randomized controlled trial

**DOI:** 10.12688/f1000research.110851.2

**Published:** 2022-07-05

**Authors:** Prasanna Mithra, Bhaskaran Unnikrishnan, Rekha T, Nithin Kumar, Ramesh Holla, Priya Rathi

**Affiliations:** 1 Department of Community Medicine, Kasturba Medical College, Mangalore, Manipal Academy of Higher Education, Manipal, India; 2 Novo Nordisk, Raipur, Chhattisgarh State, 492001, India

**Keywords:** Paternal, IYCF, involvement, module, trial

## Abstract

**Background: **Overall child health depends on nutrition and its related practices. At the family level, responsibility of child feeding lies with both parents. There is no uniform and systematic way to determine and assess the practices of fathers in infant and young child feeding (IYCF). Also, there is a paucity of evidence related to interventions for fathers in improving their practices and involvement in the feeding of their infant or young child (aged less than two years).

**Methods**: This was a community-based randomized control trial, conducted among 120 fathers with infants and/or young children in Dakshina Kannada District of Karnataka. Fathers with poor level of involvement and practices towards IYCF, during the initial assessment, were included as the study participants. For the intervention, a module in the flipchart format was developed. Simple randomization technique was used to allot the participants into two groups - intervention and control. Participants in the intervention group received module intervention, in addition to the care which they received routinely, and the control group received only routine care. The participants in the intervention group were paid a monthly visit to implement the module, for six months. The post-intervention assessment was done at the end of 6 months.

**Results**: A total of 117 participants provided post-intervention data. The mean age was 34.7 (+/- 5.48) years in the intervention group and 34.36 years (+/- 5.26) in the control group. The intervention group showed significantly higher improvement in knowledge, attitude, and practice components at 6 months (p<0.05), in both unadjusted and adjusted models.

**Conclusions**: The extent of increase in practice and involvement in child feeding was clearly higher among the intervention group. The module developed was successful in improving the practices of fathers in feeding their infants and young children.

**Clinical Trials Registry India:** CTRI/2017/06/008936 (29/06/2017)

## Introduction

The health of the children reflects the overall health of a community and country. However, child health is affected and influenced by several factors such as nutrition and feeding practices in the early months of life. These are also important determinants of children’s health, growth, and survival. ^1^^,^^2^ Although the responsibility of a child’s feeding lies with both the parents, much of the emphasis and stress has been on mothers. Several cultural and traditional factors restrict or limit the participation from the fathers. When both parents put their efforts together for the child’s feeding practices, the child has a higher chance of receiving better nutrition. ^3^^–^^6^

The important role and influence that fathers can have in shaping their children’s eating patterns have been documented. ^7^ Paternal feeding practices towards their young children also reflect the way of time management done by the fathers. ^8^ Despite this established paternal role in child-rearing, most of the research and policies related to child feeding, have not given importance to fathers. ^9^^,^^10^ Also, in real life situations, occupation or other family related factors make the fathers skip major aspects of childcare, especially feeding practices. Several strategies using media, healthcare providers, and peer-to-peer interventions have been implemented to improve child nutrition. However, all these were keeping mothers as main recipients. ^11^^,^^12^ But support from the father of the child, coupled with a change in attitude and knowledge even at the stage of breastfeeding has given promising results in terms of the success of breastfeeding and overall nutrition of the child. ^11^^,^^13^ However, in many instances, fathers possess the knowledge and positive attitude towards infant and young child feeding (IYCF), but it may not be reflected in their practices. ^6^ Thus fathers of infants and young children need a reinforced information and communication in this regard.

Many factors have a role to play in determining the level and extent of paternal practices in infant and child feeding practices. Still, there is a paucity of evidence related to these practices. ^14^^–^^17^ A simple, easily accessible and repeatable module for fathers would stimulate them to have better participation in the care of their infant or young child. This also could reinforce important factors which need special attention, like choosing weaning food, enhancing the consistency of food items as the child grows and maintenance of hygiene. Given the culturally diverse Indian population, no modular interventions for fathers to improve their IYCF practices have been evaluated so far.

Thus, this trial was carried out to develop and test the effectiveness of a module, targeting fathers with either infants or young children aged less than 2 years, in improving their involvement in IYCF.

## Methods

### Ethical issues

Approval was obtained from the Institutional Ethics Committee (IEC) of Kasturba Medical College, Mangalore (approval number IEC KMC MLR 06-17/111 dated 21 ^st^ June 2017). After obtaining the approval, data collection commenced. A participant information letter was provided and written informed consent was taken from each participant, after assuring anonymity and confidentiality for the information they provided. This article is reported in line with the Consolidated Standards of Reporting Trials (CONSORT) guidelines. ^18^

### Trial design

This community-based open-label randomized controlled trial was carried out among 120 fathers of infants and young children aged 12 to <24 months in Dakshina Kannada District (Karnataka State), India, between January and December 2020. The sample size calculation, recruitment of study participants, sampling techniques followed, methodology of randomization, intervention procedures have been described earlier. ^19^ The Template for Intervention Description and Replication (TiDier) was used for the description of the methodology. ^20^ Sample size for this trial was calculated using an assumption of 15% improvement in the paternal practices related to IYCF after module-based intervention, 80% power, 5% alpha-error, 1:1 allocation and adding 20% non-response error, as 60 participants in each group.

The eligible participants for this trial were selected using stratified multistage random sampling technique; wherein we did the stratification at subdistrict level. At each stratum, the health centers of the public health system were chosen using simple random sampling done with the help of lottery technique, with probability proportionate to size, keeping the population covered by the centres as benchmark. Each center caters to several wards (administrative sub-unit) and again using lottery technique, a ward was selected from the health center area. From the health centers, we could collect the list of infants and young children in selected wards and visit their fathers who were selected using convenient sampling method, on a pre-approved, mutually convenient time in their houses. A total of 450 eligible fathers were assessed. ^21^ The registration of this trial was done in Clinical Trials Registry- India (CTRI/2017/06/008936) on 29 June 2017. ^22^

### Participants

The fathers with at least one infant or a child aged less than 2 years were eligible for inclusion in the initial assessment. After the initial assessment of the eligible fathers in their involvement in IYCF, ^21^ they were classified as having either good or poor involvement in IYCF. This assessment included the knowledge, attitude and practice aspects of IYCF, in the form of an investigator administered questionnaire. ^18^ In case of participants with more than 1 child, the domains were assessed as applicable to the youngest child. The questionnaire consisted of scores for each of the three above mentioned domains as five-point likert scale and the total scores for practice domain were computed. Since there is no pre-existing cut-off for the score, we chose to use the median score as the cut-off to determine the number of fathers with less than 50 ^th^ centile practice score to depict poor involvement.

Those fathers with poor involvement were the eligible participants for this trial. Their list was arranged according to the Taluks (a sub-District administrative unit in India) of their residence and simple random sampling was used to select the Taluks. The participants from each of these Taluks were selected using simple random sampling. A total of 130 eligible participants were approached to meet the target sample size of 120. They were visited and explained about the objectives and nature of the intervention in the local language. In total, 10 eligible participants declined the invitation. Written informed consent was taken from all the participants who agreed to be part of this trial. The selected fathers were randomized into intervention & control groups. We used simple randomization technique with lottery method, using Microsoft Excel software. The generated sequence was converted to 120 opaque containers and arranged by principal investigator (PM). Another author (NK) performed sequential enrolment of participants. Following this, third author (RT) ensured the assignment of participants to the appropriate interventions. Final implementation of the intervention was done by research assistants.

### Outcomes

Baseline and post-intervention assessments at the end of 6 months were done using a content validated questionnaire, which included demographic component, awareness, attitude, and practices related to IYCF. This questionnaire was developed by the authors and tested for internal consistency along with inter-rater reliability. We used Cronbach alpha for internal consistency and correlation for validity for the domains of knowledge, attitude and practices. Minor revisions were then done to reach acceptable level of reliability. The questionnaire and other intervention materials can be found as *Extended data* ^18^) At the end of 6 months, due to the coronavirus disease 2019 (COVID-19) pandemic lockdown situation, the post-intervention assessment was conducted over the telephone and google form-based blended approach due to the inability to travel to the field and to avoid personal contact with the participants.

### Intervention procedure

For the intervention arm, the IYCF module was developed towards the improvement of paternal involvement in IYCF, as a pictorial-explanation flip-chart form based on extensive literature review and existing knowledge in the field. ^18^ The module included information on feeding the baby from the time of birth, ways in which a father can support the mother in breastfeeding, significance of paternal decision in child feeding practices, when and how weaning has to be started for the baby and ways of father’s involvement in general childcare. This information was in the form of bulleted points and supported by relevant images. For e.g., exclusive breast feeding and how fathers can help the mothers in this practice; food items not recommended for infant feeding; food items to be included during weaning etc. The IYCF module was implemented in Kannada (the local language) after it was pilot tested for operational feasibility. Pilot testing was done in the field among five fathers with poor involvement in IYCF, but not part of the main trial. The interactions were documented, difficulties in communications, usage of terms were addressed. Also, feedback was taken from the participants. Finally, revision of the module was undertaken.

**Figure 1.**
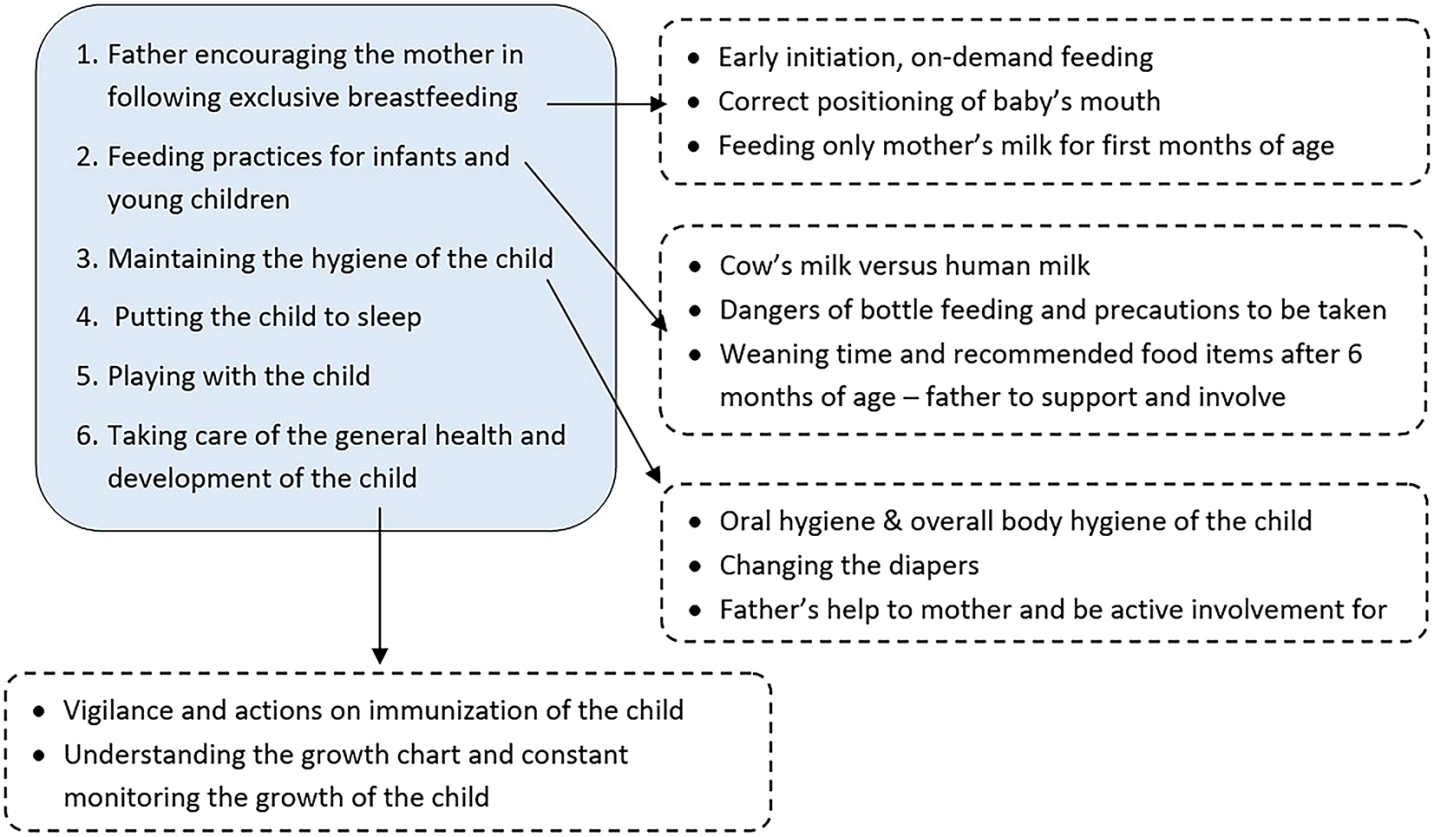
IYCF module components.

The intervention was done by two research assistants from the Medical Social Work background, who were trained in implementing the module in the community. They visited the participants on pre-informed dates at their homes or a convenient location and at a convenient time. The module in the flip-chart mode was implemented to the intervention arm on a one-to-one interaction basis. Figure 1 depicts the components and important aspects addressed in the module. The participants had a look at the pictures in the flip-chart to start the interaction, accompanied by the explanations by research assistant. This was followed by the rotation of the flip chart to get the view of the same points in sentence format. This process was done for all components of the module. Each interaction lasted for 20 to 30 minutes including the question-and-answer sessions. During this interaction, only the participant father was present along with the research assistant, to avoid any intervention contamination. The administration of the same module was done once a month for 6 months. Participants in the control group received their regular care in the health centers, whenever they visited with their child and during regular visits to them by the health workers.

### Data analysis

We analyzed the data using IBM SPSS Statistics for Windows, Version 25.0 (RRID:SCR_016479). Data management was done at the central coordinating site, located in the study institute. Results were arranged as proportions, mean (with standard deviation) using the tables. Individual scores of knowledge, attitude and practice components were added from the Likert scale response of each component and a total score was obtained. These total scores were used to compare the intervention and control groups.

The comparison for the knowledge, attitude, and practice scores across the groups was done using chi-square test, paired & independent sample ‘t’ tests. Intention to treat analysis was followed. Imputation method was used to derive the missing variables for lost to follow up participants. Cohen’s ‘d’ (with 95% confidence interval) was calculated to estimate the effect size for the intervention. An estimate of more than 0.8 was considered as large effect. For the comparison of effects of intervention on final outcomes after adjusting for covariates spread unequally across intervention and control arms, mixed effects linear regression model was used. The models were constructed with knowledge, attitude and practice post intervention scores as dependent variables, each with fixed effects of time (baseline), age and education, along with random effects of occupation and baseline knowledge. Also, the interaction of these covariates on the outcome was assessed. For mixed effects linear model, F value and partial eta square (η ^2^) were reported. A p-value <0.05 was considered statistically significant.

## Results

**Figure 2.**
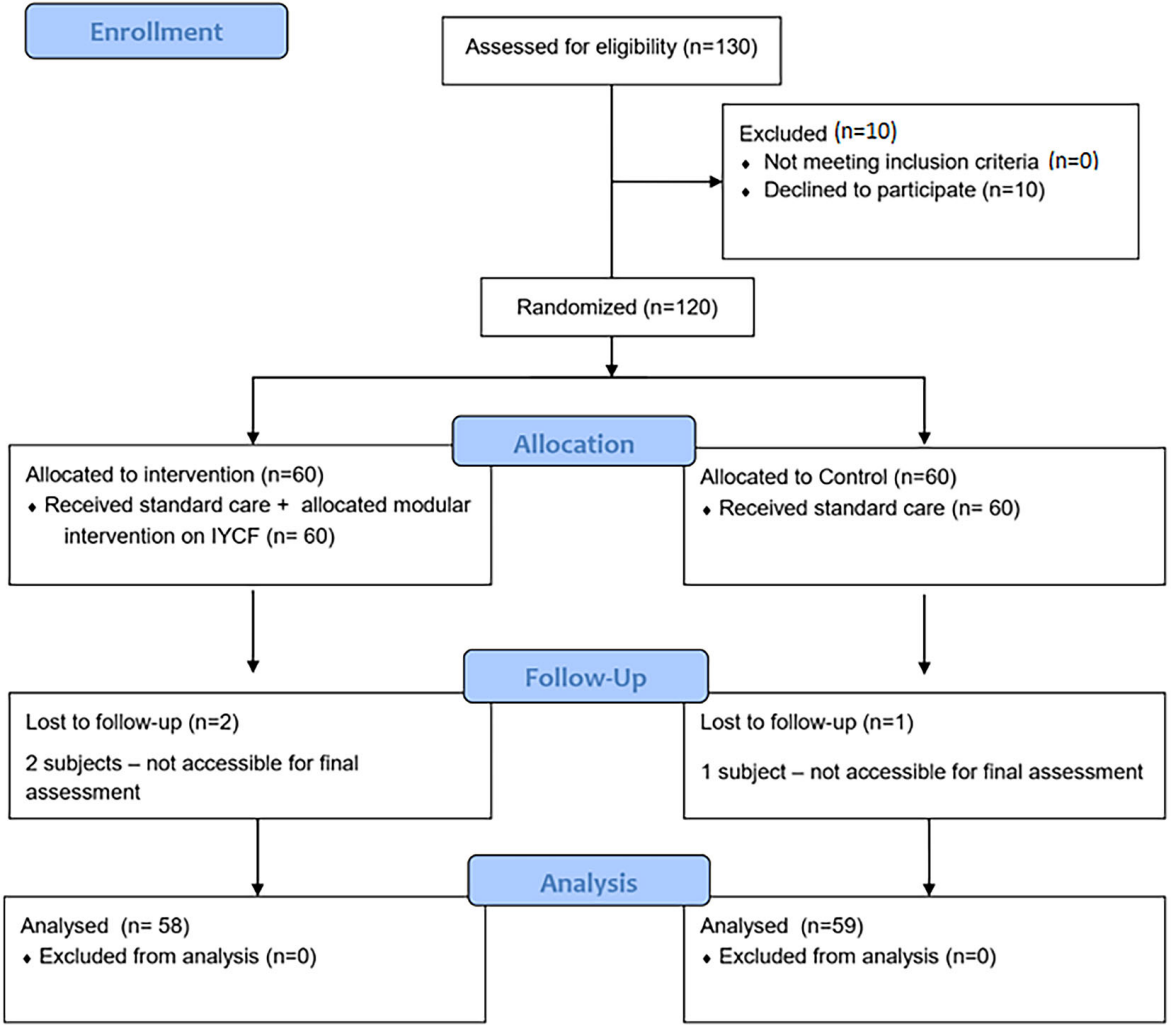
Study CONSORT diagram. IYCF=Infant and Young Child Feeding.

In total, 130 fathers were assessed for eligibility of which 120 were randomized. All the selected participants who were randomized into the intervention arm, attended one session per month for six months to complete the modular interventions. However, for the post intervention outcome assessment, two participants in the intervention and one in the control group could not be accessible after several attempts to provide follow up information on IYCF practices. Finally, 117 participants (n=58 for intervention, n=59 for control) completed the outcome assessment. ^18^ The flow of study as per the CONSORT format is provided in Figure 2. ^23^^,^^24^

The mean age of the intervention group was 34.7 (±5.48) years and the control group was 34.36 years (±5.26) (p=0.711). Both the study groups were similar concerning their demographic characteristics (age group, educational status, being first-time father or experienced father, attitude, and practice scores). The two groups were significantly different concerning the occupation categories and knowledge scores (p=0.03 and <0.0001, respectively) at the baseline ( Table 1).

**Table 1.** Baseline characteristics of the study population (N=120).

Characteristics		Intervention group (n=60) No. (%)	Control group (n=60) No. (%)	p-value
**Age group (years)**	</=30	13 (21.7)	19 (31.7)	0.49
	31-40	38 (63.3)	34 (56.7)	
	>/-41	09 (15.0)	07 (11.6)	
**Educational status**	Less than high school	23 (38.3)	29 (48.3)	0.39
	Completed high school	22 (36.7)	14 (23.3)	
	Pre-degree	06 (10.0)	07 (11.7)	
	Graduation and above	09 (15.0)	10 (16.7)	
**Occupation**	Unskilled	07 (11.6)	18 (30.0)	0.03 *
	Semiskilled	13 (21.7)	16 (26.7)	
	Skilled	31 (51.7)	22 (36.7)	
	Professional	09 (15.0)	04 (06.6)	
**Parenthood**	First-time fathers	24 (40.0)	32 (53.3)	0.16
	Experienced fathers	36 (60.0)	28 (46.7)	

		Mean (SD ^#^)	Mean (SD ^#^)	
**Age (Years)**		34.70 (5.48)	34.36 (5.26)	0.71
**Knowledge score**		55.35 (6.58)	60.03 (2.28)	<0.0001 *
**Attitude score**		15.85 (2.46)	16.37 (1.29)	0.19
**Practice score**		30.95 (1.35)	30.71 (3.89)	0.39

* p-value significant at 0.05 level.

^#^ SD=Standard Deviation.

The before and after comparisons of the scores are described in Table 2. Post intervention mean scores were adjusted for occupation and baseline knowledge scores which varied across intervention and control arms. The intervention group showed statistically significant (p<0.0001) improvement in all three scores, i.e., knowledge, attitude and practice scores. In the control group, there was a decline in the knowledge scores at 6 months. We found this difference to be statistically significant (p<0.0001).

**Table 2.** Comparison between baseline and post-intervention scores across the study groups.

	Intervention group (n=60)				Control group (n=60)			
	Baseline Mean (SD)	Post-intervention Mean (SD) (Unadjusted)	Post-intervention Mean (SD) (Adjusted)	p-value	Baseline Mean (SD)	Post-intervention Mean (SD) (Unadjusted)	Post-intervention Mean (SD) (Adjusted)	p-value
**Knowledge score**	55.35 (6.58)	80.32 (2.30)	80.95 (1.11)	<0.0001 *	60.03 (2.28)	52.43 (9.88)	51.78 (0.99)	<0.0001 *
**Attitude score**	15.85 (2.46)	18.24 (1.29)	18.37 (0.22)	<0.0001 *	16.37 (1.29)	16.99 (1.86)	16.89 (0.22)	.061
**Practice score (involvement in IYCF)**	30.95 (1.35)	49.80 (4.10)	50.01 (0.96)	<0.0001 *	30.71 (3.89)	34.25 (8.57)	33.97 (0.94)	.005 *

* p-value significant at 0.05 level.

^#^ SD=Standard Deviation, IYCF=infant and young child feeding.

The mixed effects linear regression model showed significant treatment effect for knowledge, (F _(1, 113)_=59.6, η ^2^=0.35, p<0.0001), attitude F _(1, 113)_=11.1, η ^2^=0.9, p=0.001) and practice (IYCF involvement) (F _(1, 114)_=16.68, η ^2^=0.13, p<0.0001) across the intervention and control arms. (η ^2^ – partial eta squared). However, the covariates – occupation of the participants and baseline knowledge on IYCF had no significant main effects on any of the three domains assessed. Also the interaction of these covariates was not found to be statistically significant. On knowledge, the interaction was - F _(2, 113)_=0.33, p=0.72, on attitude - F _(2, 113)_=1.64, p=0.98 and on practice – F _(2, 114)_=0.23, p=0.79.

There was also improvement in the attitude; however, the difference was not found to be statistically significant (p=0.061). Also, there was a statistically significant improvement in practice scores in the control group (p=0.005). When the change scores were compared across the two groups between pre-intervention and post-intervention scores, all the three change scores (knowledge, attitude and practice) were higher for the intervention group. These differences were found to be statistically significant (p<0.0001). The details of the change score comparisons are given in Table 3. The correlation test between change scores of knowledge and practice aspects of IYCF revealed significant association. Further, the estimation of effect size (Cohen’s ‘d’) revealed large effects for the change scores of all the three components across the intervention arms. The estimate for knowledge domain was 1.75 (95% CI: 1.57-1.92), attitude 0.65 (95% CI: 0.31-0.99) and practice domain 1.54 (95% CI: 1.32-1.78).

**Table 3.** Comparison of change scores across the two study groups (N=117).

	Intervention group Mean (SD)	Control group Mean (SD)	p-value
**Knowledge score**	24.89 (0.88)	-7.61 (1.41)	<0.0001 *
**Attitude score**	02.38 (0.38)	0.61 (0.32)	0.001 *
**Practice score (IYCF involvement)**	19.10 (0.54)	3.17 (1.08)	<0.0001 *

* p-value significant at 0.05 level.

^#^ SD=Standard Deviation, IYCF=infant and young child feeding.

## Discussion

Father’s support can greatly improve the status of mother and child health in the community, by bringing positive change in feeding practices of children right from birth. ^5^ Also, the fathers’ parenting skills, feeding practices may the nutritional behaviour and overall growth and development of the child. ^9^^,^^25^^,^^26^ Although, because of several changes in society, fathers’ involvement in childcare has been witnessing an upward trend over time, it is still influenced by a multitude of factors. ^5^^,^^7^^,^^27^ Being a father is challenging in terms of overall childcare and the child’s diet. ^28^ These challenges could be in terms of influence of other elderly family members in deciding the IYCF, lack of experience in childcare, absence of felt need of their involvement and many hidden factors. Neha Khandpur *et al*, in 2014, in their systematic review, reported that feeding practices varied in mothers and fathers. Fathers’ feeding practices towards children contributed significantly to the nutritional status of children. ^9^ This review also highlighted the shortage of literature and thereby information on child feeding practices of fathers. The current situation of limited fathers’ involvement in IYCF and influencing factors have been reported in our earlier paper. ^21^ We reported a 40.9% of fathers of infants or young children having poor involvement in IYCF and the same was higher among fathers belonging to urban area. Younger age and having education above graduation was associated with better involvement in IYCF. Occupation did not have a direct influence on involvement of fathers in IYCF. Also, these fathers had favourable attitude towards receiving education and training on handling babies and children, so that overall growth of their children would not be compromised.

Better paternal involvement in IYCF is possible with provision of adequate knowledge to the fathers of young children, in addition to prospective fathers. ^27^ Considering the current Indian scenario, wherein most of the child nutrition and care-related interventions have been focusing on mothers, ^25^ we developed a unique and simple father-oriented module. ^18^ This module targeted an increase in knowledge, improvement in attitude and practices towards IYCF. As reported by Han *et al* in 2019, ^29^ behaviour change communication (BCC) strategies are important in enhancing involvement in child feeding. However, they also noted that only knowledge gain would have a limited impact on IYCF practices. They involved mothers of children along with fathers in their community-based trial.

In our trial, the group of fathers, after receiving the modular intervention in addition to the standard care, had significant improvement in knowledge, attitude and involvement towards their IYCF. Also, the control group which received standard care showed a decline in their knowledge, a mild increase in attitude, and a significant increase in practice. However, the change scores reflected a higher difference due to the implementation of this module. Several varieties of interventions have been tried, both in developing and developed parts of the world. A trial by Pisacane A *et al* in Italy, conducted in 2005 towards teaching the fathers on handling and managing lactational problems showed better breastfeeding practices till 6 months. ^11^ Similarly, Han *et al* in 2019, ^29^ assessed the impact of both-parent behavior change programs through communication on IYCF practices compared to the maternal program alone. They noted that father’s IYCF knowledge increase was highest when it was clubbed with maternal BCC as compared to paternal BCC alone. However, they also reported that additional gain in knowledge did not translate to further enhancement in practices of IYCF. Elizabeth Sloand *et al* in Haiti ^30^ evaluated a public health nursing strategy using village-based fathers clubs. The post-intervention opinion from the participants revealed that childcare and health improved because of this intervention. A quasi-experimental study by Faith Thuita *et al*, in Kenya, in 2015, studied the impact of involving fathers and/or grandmothers on diets of mothers and IYCF practices in a rural setting and they found this model successful. ^12^

There have also been attempts to enhance paternal involvement in IYCF, right from the antenatal period of their spouses. Jenny Tohotoa *et al* in Perth, Australia, in 2009, carried out antenatal sessions for fathers from different socio-economic strata and reported that a father-oriented approach was successful in terms of acceptance of the education material and involvement in baby feeding following the delivery of their spouse. ^28^ Bruce Maycock *et al* in Australia, in their fathers infant feeding initiative (FIFI Study), provided antenatal education sessions and postnatal support targeted to fathers and they reported that higher age and socioeconomic status of fathers had better breastfeeding rates. ^31^

Another quasi-experimental study carried out by Abdullahi *et al*, in Somalia, in 2019, aimed at assessing the peer counselling effects by either mother or father as a support group on IYCF practices. They noticed a positive trend in knowledge, breastfeeding practices, and diet diversity among intervention arms. ^32^ Shorey *et al* in Singapore in 2017, noted that enforcement of paternal involvement throughout the perinatal period by healthcare professionals had a positive response. ^17^

In our study, we also noted that knowledge and practice scores showed higher change scores as compared to attitude scores in both the study arms. This could be due to pre-existing higher baseline scores related to attitude. However, the intervention arm has higher change scores. This could be further supported by the observation made in study which noted the decline in knowledge of control arm, which had a higher knowledge score at the baseline than intervention arm. All the previous studies showed improvement in paternal practices towards IYCF to a variable extent. However, the applicability to the wider population, the need for a professional to provide the interventions, and accessing the fathers at the right time have been some of the challenges faced by these studies. But the current module has been made simple to use by a grass-root level health worker and is made culturally neutral. Similar modular intervention-based approaches could be implemented in wider populations. The current study was carried out in a District which is having high literacy rates and better health-seeking behaviors. Thus, further effects in other regions of the country and outside regions remain to be tested. Also, the long-term effect of this module intervention, which could not be assessed in this study, would signify the need for re-enforcement of relevant information on paternal involvement in IYCF. Consequently, it would benefit the health care practitioners and policymakers in improving child growth and development through the strengthening of paternal involvement in IYCF and suitably planned interventions.

## Conclusions

The participating intervention and control groups were similar to each other concerning most of the demographic characteristics. Both the groups had significant improvement in attitude and practice components at the end of 6 months, but the change scores were significantly higher for the intervention group. Also, concerning knowledge related to IYCF, the control group had a decline, and the intervention arm showed significant improvement. Thus, the module developed was successful in improving the overall involvement of fathers in their infant and young child feeding.

## Data availability

### Underlying data

Open Science Framework: Effectiveness of a module-based intervention on paternal involvement in Infant and young child feeding (IYCF) practices in Coastal South India - A Randomized Controlled Trial. https://doi.org/10.17605/OSF.IO/D9GZ5. ^18^

This project contains the following underlying data:

- RCT final with all raw data.xlsx (this dataset includes the compiled scores in knowledge, attitude and practice domains at baseline and post-intervention; 1A1-1C14 reflect pre and 2A1-2C14 reflect post intervention data)

### Extended data

Open Science Framework: Effectiveness of a module-based intervention on paternal involvement in Infant and young child feeding (IYCF) practices in Coastal South India - A Randomized Controlled Trial. https://doi.org/10.17605/OSF.IO/D9GZ5. ^18^

This project contains the following extended data:

- CONSORT-Final-RCT.png (CONSORT flowchart)
- Informed consent-F1000.docx (Participant information sheet and consent form)
- IYCF module.pdf (intervention material)
- Questionnaire-F1000.docx

### Reporting guidelines

Open Scientific Framework: CONSORT checklist ^33^ for ‘Module intervention to improve involvement and practices of fathers towards infant and young child feeding (IYCF) in Coastal South India - a randomized controlled trial’. https://doi.org/10.17605/OSF.IO/D9GZ5. ^18^

Data are available under the terms of the Creative Commons Attribution 4.0 International license (CC-BY 4.0).
