# Supplementary material for: Module intervention to improve involvement and practices of fathers towards infant and young child feeding (IYCF) in Coastal South India - a randomized controlled trial
Source: F1000Res. 2022 Jul 5;11:486. Originally published 2022 May 3. [Version 2] doi: 10.12688/f1000research.110851.2 (PMC9277194; doi:10.12688/f1000research.110851.2)
Supplement: Peer review history

# Peer review: Reviewer response for version 2

**DOI:** 10.5256/f1000research.135364.r143259

**Published:** 2022-07-12

**Authors:** Sathish Thirunavukkarasu

**Affiliations:** 1 Dept. of Family and Preventive Medicine, Emory University, Atlanta, GA, USA

Thank you authors for addressing my comments satisfactorily.

Is the work clearly and accurately presented and does it cite the current literature?

Yes

If applicable, is the statistical analysis and its interpretation appropriate?

No

Are all the source data underlying the results available to ensure full reproducibility?

Yes

Is the study design appropriate and is the work technically sound?

Yes

Are the conclusions drawn adequately supported by the results?

Yes

Are sufficient details of methods and analysis provided to allow replication by others?

Yes

Reviewer Expertise:

NA

I confirm that I have read this submission and believe that I have an appropriate level of expertise to confirm that it is of an acceptable scientific standard.

# Peer review: Reviewer response for version 2

**DOI:** 10.5256/f1000research.135364.r143258

**Published:** 2022-07-12

**Authors:** Amutha Ramadas

**Affiliations:** 1 Jeffrey Cheah School of Medicine and Health Sciences, Monash University Malaysia, Bandar Sunway, Malaysia

I am satisfied with the changes made to the paper based on previous feedback.

Is the work clearly and accurately presented and does it cite the current literature?

Yes

If applicable, is the statistical analysis and its interpretation appropriate?

Partly

Are all the source data underlying the results available to ensure full reproducibility?

No

Is the study design appropriate and is the work technically sound?

Yes

Are the conclusions drawn adequately supported by the results?

Partly

Are sufficient details of methods and analysis provided to allow replication by others?

Partly

Reviewer Expertise:

Nutrition

I confirm that I have read this submission and believe that I have an appropriate level of expertise to confirm that it is of an acceptable scientific standard.

# Peer review: Reviewer response for version 2

**DOI:** 10.5256/f1000research.135364.r143257

**Published:** 2022-07-07

**Authors:** Elezebeth Mathews

**Affiliations:** 1 Department of Public Health and Community Medicine, Central University of Kerala, Kasaragod, Kerala, India

I have reviewed the manuscript. Authors have addressed the comments and articulated it satisfactorily in the manuscript. The version 2 of the manuscript may be accepted for indexing.

Is the work clearly and accurately presented and does it cite the current literature?

Yes

If applicable, is the statistical analysis and its interpretation appropriate?

Yes

Are all the source data underlying the results available to ensure full reproducibility?

Yes

Is the study design appropriate and is the work technically sound?

Yes

Are the conclusions drawn adequately supported by the results?

Yes

Are sufficient details of methods and analysis provided to allow replication by others?

Yes

Reviewer Expertise:

NA

I confirm that I have read this submission and believe that I have an appropriate level of expertise to confirm that it is of an acceptable scientific standard.

# Peer review: Reviewer response for version 1

**DOI:** 10.5256/f1000research.122505.r136588

**Published:** 2022-06-15

**Authors:** Amutha Ramadas

**Affiliations:** 1 Jeffrey Cheah School of Medicine and Health Sciences, Monash University Malaysia, Bandar Sunway, Malaysia

Overall, the manuscript is clearly written for comprehension of the scientific community. The title and abstract reflected the content of the manuscript. 

 I believe the article may benefit from the following input:

1. Introduction is brief and should provide more in-depth background info and justification for the research gap. 
2. Methods - The flow is clear, and ethics approval and sample size justification have been provided. However the intervention may need to be described further, perhaps with some examples of the materials used. 
3. Results - I have a concern with the significant difference in occupation status between groups, which could have impacted the results but was not adjusted for when the main outcomes were compared.

Is the work clearly and accurately presented and does it cite the current literature?

Yes

If applicable, is the statistical analysis and its interpretation appropriate?

Partly

Are all the source data underlying the results available to ensure full reproducibility?

No

Is the study design appropriate and is the work technically sound?

Yes

Are the conclusions drawn adequately supported by the results?

Partly

Are sufficient details of methods and analysis provided to allow replication by others?

Partly

Reviewer Expertise:

Nutrition

I confirm that I have read this submission and believe that I have an appropriate level of expertise to confirm that it is of an acceptable scientific standard, however I have significant reservations, as outlined above.

# Response

**Published:** 2022-06-20

**Authors:** Prasanna Mithra

**Affiliations:** Kasturba Medical College, Mangalore, India

Reviewer Comments; followed by the response (in bold) by Authors:

 Overall, the manuscript is clearly written for comprehension of the scientific community. The title and abstract reflected the content of the manuscript. I believe the article may benefit from the following input:

 Introduction is brief and should provide more in-depth background info and justification for the research gap.

**Thank you for the valuable input and suggestions. The “Introduction” section is now revised to add relevant points as suggested and background information is made more robust. A stronger justification for the research gap is now added.**

 Methods - The flow is clear, and ethics approval and sample size justification have been provided. However the intervention may need to be described further, perhaps with some examples of the materials used.

**Thank you. We have now revised the text related to the intervention procedure. Have added more details on the module and added examples at relevant places as per the suggestion.**

 Results - I have a concern with the significant difference in occupation status between groups, which could have impacted the results but was not adjusted for when the main outcomes were compared.

**Thank you for the suggestion. We have now relooked into the analysis part based on the comments of the reviewers and have adjusted for the baseline differences to report the outcomes. Both the adjusted estimates and effect of interactions have been reported.**

# Peer review: Reviewer response for version 1

**DOI:** 10.5256/f1000research.122505.r136587

**Published:** 2022-05-24

**Authors:** Elezebeth Mathews

**Affiliations:** 1 Department of Public Health and Community Medicine, Central University of Kerala, Kasaragod, Kerala, India

Authors have rightly identified the research gap of paternal involvement in child care and nutrition. This was a randomized controlled trial to enhance the involvement of fathers in infant and young child feeding practices among those fathers identified to have poor involvement in child care in Dakshina Kannada District. The authors have described the methodology clearly in this manuscript as deemed necessary and included citations for further details. This is a well-written manuscript.

 There are no major revisions required, however, the details of the tests for validity and reliability of the outcome measurement tool need to be mentioned. The intervention procedure may be explained further in terms of intervention material and delivery. Though what constitutes the flipchart is mentioned, a table or a figure with the contents of each module in English Language will be useful. 

 In the results section, please mention if all participants attended six sessions (once per month for six months). The baseline characteristics were similar for both the groups except for the occupation. Authors may adjust for the inherent occupational differences in the intervention and control arm for the outcome. Consider doing a statistical analysis wherein you estimate the effect size.

Is the work clearly and accurately presented and does it cite the current literature?

Yes

If applicable, is the statistical analysis and its interpretation appropriate?

Yes

Are all the source data underlying the results available to ensure full reproducibility?

Yes

Is the study design appropriate and is the work technically sound?

Yes

Are the conclusions drawn adequately supported by the results?

Yes

Are sufficient details of methods and analysis provided to allow replication by others?

Yes

Reviewer Expertise:

Behavioural science, chronic disease epidemiology

I confirm that I have read this submission and believe that I have an appropriate level of expertise to confirm that it is of an acceptable scientific standard, however I have significant reservations, as outlined above.

# Response

**Published:** 2022-06-20

**Authors:** Prasanna Mithra

**Affiliations:** Kasturba Medical College, Mangalore, India

Reviewer Comments; followed by the response (in bold) by Authors:

 Authors have rightly identified the research gap of paternal involvement in child care and nutrition. This was a randomized controlled trial to enhance the involvement of fathers in infant and young child feeding practices among those fathers identified to have poor involvement in child care in Dakshina Kannada District. The authors have described the methodology clearly in this manuscript as deemed necessary and included citations for further details. This is a well-written manuscript.

 There are no major revisions required, however, the details of the tests for validity and reliability of the outcome measurement tool need to be mentioned. The intervention procedure may be explained further in terms of intervention material and delivery. Though what constitutes the flipchart is mentioned, a table or a figure with the contents of each module in English Language will be useful.

**Thank you for your valuable suggestions and comments. We have now added detail on the test used for the reliability and validity of the data collection tool. We have now added a pictorial component describing the module content briefly. Also we have described the module delivery process for more clarity.**

 In the results section, please mention if all participants attended six sessions (once per month for six months).

**We have added a sentence mentioning all participants having attended the sessions once a month for 6 months.**

 The baseline characteristics were similar for both the groups except for the occupation.

 Authors may adjust for the inherent occupational differences in the intervention and control arm for the outcome.

**Thank you. We have now revised the statistical methods used for analyzing the effect of interventions and adjusted outcomes have been reported. We have used a linear mixed-effects regression model. The baseline differences were included in the model.**

 Consider doing a statistical analysis wherein you estimate the effect size.

**Thank you for the suggestion. We have now done the analysis and estimated the effect size. The same has been mentioned in the data analysis description.**

# Peer review: Reviewer response for version 1

**DOI:** 10.5256/f1000research.122505.r136589

**Published:** 2022-05-23

**Authors:** Sathish Thirunavukkarasu

**Affiliations:** 1 Dept. of Family and Preventive Medicine, Emory University, Atlanta, GA, USA

This article by Mithra et al. examined the effectiveness of a module intervention in improving the involvement and practices of 120 fathers towards infant and young child feeding in a district in Karnataka state, India. The manuscript addresses an interesting topic, and it is well written. However, as detailed below, I have some significant concerns about the statistical methods used for analyses.

1. Provide information on blinding participants and others involved in the trial, including those who conducted the analysis.
2. Three participants were lost to follow-up, and leaving them out of the analysis is not an intention-to-treat (IIT) analysis. I agree it is a tiny number, but it would be good to impute the missing data using some ideal techniques to satisfy the IIT principle.
3. The authors did not use appropriate statistical methods for the analyses. Paired 't' test does not consider the correlation between repeated measurements taken on the same individual over time and does not adjust for any baseline differences in covariates. In this study, occupation and the knowledge score were not balanced between the two arms (please don't rely on the statistical tests for baseline differences), and they were not adjusted for in the analyses. Finally, with 't' test one cannot say that the mean change in one arm was higher or lower than that in the other arm (which the authors claim). I suggest using mixed-effects linear regression models for the analyses.
4. Was there a possibility of contamination between study arms? If yes, how was it handled?
5. Why did the attitude score in the intervention group show lesser improvement than the knowledge and practice scores? Please provide some insights on this.

Is the work clearly and accurately presented and does it cite the current literature?

Yes

If applicable, is the statistical analysis and its interpretation appropriate?

No

Are all the source data underlying the results available to ensure full reproducibility?

Yes

Is the study design appropriate and is the work technically sound?

Yes

Are the conclusions drawn adequately supported by the results?

Yes

Are sufficient details of methods and analysis provided to allow replication by others?

Yes

Reviewer Expertise:

Chronic disease epidemiology, particularly diabetes.

I confirm that I have read this submission and believe that I have an appropriate level of expertise to state that I do not consider it to be of an acceptable scientific standard, for reasons outlined above.

# Response

**Published:** 2022-06-20

**Authors:** Prasanna Mithra

**Affiliations:** Kasturba Medical College, Mangalore, India

Reviewer Comments; followed by the response (in bold) by Authors:

 This article by Mithra et al. examined the effectiveness of a module intervention in improving the involvement and practices of 120 fathers towards infant and young child feeding in a district in Karnataka state, India. The manuscript addresses an interesting topic, and it is well written. However, as detailed below, I have some significant concerns about the statistical methods used for analyses.

 Provide information on blinding participants and others involved in the trial, including those who conducted the analysis.

**Thank you for your valuable suggestions and review. It was an open-label intervention. The nature of the intervention was not conducive to blind the participants or investigators. We have now specified the same in methodology.**

 Three participants were lost to follow-up, and leaving them out of the analysis is not an intention-to-treat (IIT) analysis. I agree it is a tiny number, but it would be good to impute the missing data using some ideal techniques to satisfy the IIT principle.

**We have now used imputation for missing numbers and carried out ITT for all the outcome variables. A sentence is now added in this regard in the data analysis section. Data reanalysis is done and reported in the tables.**

 The authors did not use appropriate statistical methods for the analyses. Paired 't' test does not consider the correlation between repeated measurements taken on the same individual over time and does not adjust for any baseline differences in covariates. In this study, occupation and the knowledge score were not balanced between the two arms (please don't rely on the statistical tests for baseline differences), and they were not adjusted for in the analyses. Finally, with 't' test one cannot say that the mean change in one arm was higher or lower than that in the other arm (which the authors claim). I suggest using mixed-effects linear regression models for the analyses.

**Thank you for the valuable suggestions and input. We have redone the data analysis using the mixed-effects linear model and reported the findings, to take care of the baseline differences across both arms. Also, we have now reported the adjusted mean scores for all the three outcome variables (adjusted for occupation and baseline knowledge scores). These have been now added to Table 2 to keep up the comprehensive structure and also to make it complete.**

 Was there a possibility of contamination between study arms? If yes, how was it handled?

**Thank You. We chose the study areas for interventions from different taluks, to avoid contamination.**

 Why did the attitude score in the intervention group show lesser improvement than the knowledge and practice scores? Please provide some insights on this.

**Both the groups had a higher attitude score in the baseline itself, so it showed the ceiling limit effect. A brief description in this regard has been added in the discussion section with an explanation for the same.**
